# Evaluating the Efficacy of Pre-Emptive Peribulbar Blocks with Different Local Anesthetics or Paracetamol Using the Adequacy of Anesthesia Guidance for Vitreoretinal Surgeries: A Preliminary Report

**DOI:** 10.3390/biomedicines12102303

**Published:** 2024-10-10

**Authors:** Michał Jan Stasiowski, Anita Lyssek-Boroń, Katarzyna Krysik, Dominika Majer, Nikola Zmarzły, Beniamin Oskar Grabarek

**Affiliations:** 1 Chair and Department of Emergency Medicine, Faculty of Medical Sciences in Zabrze, Medical University of Silesia, 40-055 Katowice, Poland; 2 Department of Ophthalmology, St. Barbara Hospital, Trauma Centre, 41-200 Sosnowiec, Poland; anitaboron3@gmail.com (A.L.-B.); kkrysik@gmail.com (K.K.); 3 Department of Ophthalmology, Faculty of Medicine, Academy of Silesia, 40-555 Katowice, Poland; 4 Department of Anaesthesiology and Intensive Care, St Barbara’s 5th Regional Hospital, Trauma Centre, 41-200 Sosnowiec, Poland; lekd.niedziela@gmail.com; 5 Department of Ophthalmology, Prof. Kornel Gibiński Memorial University Clinical Centre, Medical University of Silesia, 40-752 Katowice, Poland; 6 Collegium Medicum, WSB University, 41-300 Dabrowa Gornicza, Poland; nikola.zmarzly@gmail.com (N.Z.); bgrabarek7@gmail.com (B.O.G.)

**Keywords:** adequacy of anesthesia, bupivacaine, intolerable postoperative pain perception, peribulbar block, ropivacaine, surgical pleth index, numeric pain rating scale, vitreoretinal surgery

## Abstract

Background/Objectives: Precisely selected patients require vitreoretinal surgeries (VRS) performed under general anesthesia (GA) when intravenous rescue opioid analgesics (IROA) are administered intraoperatively, despite a risk of adverse events, to achieve hemodynamic stability and proper antinociception and avoid the possibility of intolerable postoperative pain perception (IPPP). Adequacy of anesthesia guidance (AoA) optimizes the titration of IROA. Preventive analgesia (PA) techniques and intravenous or preoperative peribulbar block (PBB) using different local anesthetics (LAs) are performed prior to GA to optimize IROA. The aim was to analyze the utility of PBBs compared with intravenous paracetamol added to AoA-guided GA on the incidence of IPPP and hemodynamic stability in patients undergoing VRS. Methods: A total of 185 patients undergoing vitreoretinal surgery (VRS) were randomly assigned to one of several anesthesia protocols: general anesthesia (GA) with analgesia optimized through AoA-guided intraoperative remifentanil opioid analgesia (IROA) combined with a preemptive single dose of 1 g of paracetamol (P group), or PBB using one of the following options: 7 mL of an equal mixture of 2% lidocaine and 0.5% bupivacaine (BL group), 7 mL of 0.5% bupivacaine (BPV group), or 7 mL of 0.75% ropivacaine (RPV group). According to the PA used, the primary outcome measure was postoperative pain perception assessed using the numeric pain rating scale (NPRS), whereas the secondary outcome measures were as follows: demand for IROA and values of hemodynamic parameters reflecting quality or analgesia and hemodynamic stability. Results: A total of 175 patients were finally analyzed. No studied PA technique proved superior in terms of rate of incidence of IPPP, when IROA under AoA was administered (*p* = 0.22). PBB using ropivacaine resulted in an intraoperative reduction in the number of patients requiring IROA (*p* = 0.002; *p* < 0.05) with no influence on the dose of IROA (*p* = 0.97), compared to paracetamol, and little influence on hemodynamic stability of no clinical relevance in patients undergoing VRS under AoA-guided GA. Conclusions: PA using paracetamol or PBBs, regardless of LAs used, in patients undergoing VRS proved no advantage in terms of rate of incidence of IPPP and hemodynamic stability when AoA guidance for IROA administration during GA was utilized. Therefore, PA using them seems no longer justified due to the potential, although rare, side effects.

## 1. Introduction

Anesthesia for patients undergoing vitreoretinal surgeries (VRS) ranges from general anesthesia (GA) regimens [1] to regional anesthesia (RA) techniques with monitored anesthesiological care (MAC) [2,3] to combine patients’ individual needs with operators’ comfort. On the one hand, RA with MAC does not guarantee proper immobilization on the operating table in the case of some elderly patients, but on the other hand, the necessity of VRS performance under GA involves the necessity of intravenous rescue opioid analgesia (IROA) administration. IROA has been proven to trigger the incidence of adverse events such as bradycardia, hypotension, constipation, tiredness, vertigo, sleep and concentration disorders [4], and postoperative nausea and vomiting (PONV) in the case of their overdose [5], whereas its underdosing may bear responsibility for evoking intolerable postoperative pain perception (IPPP) [6] in the mechanism of central sensitization [7], observed at the rate of even up to 56% in patients undergoing VRS [8]. Further IROA dosing optimization is attempted by the addition of different preoperative preventive analgesia (PA) techniques to GA in patients undergoing VRS, either regional or intravenous. PA was proven to reduce the rate of incidence of the abovementioned adverse events [5,9,10,11], diminish the rate of oculocardiac reflex (OCR), resulting in rapid hemodynamic decompensation [5], and PONV, including the most dangerous retching, IPPP, defined as a pain perception >3, using the numerical pain rating score (NPRS 0–10), into an acceptable level. although their employment did not succeed in its complete elimination and contraindications to their use are numerous.

Therefore, accurately adjusting intraoperative rescue opioid analgesia (IROA) poses a significant challenge, requiring careful observation of fluctuations in the aforementioned hemodynamic parameters along with anesthesiologist expertise. This is particularly challenging because inadequate analgesia during surgery may not always manifest as tachycardia or hypertension, as volatile anesthetics often dampen the hemodynamic response to pain stimuli. This effect is especially pronounced in undiagnosed diabetic patients and the elderly [12]. The adequacy of anesthesia is a new concept (AoA) based on entropy EEG to separately measure and administer the hypnotic component of GA and the surgical pleth index (SPI), a new method of separate objective assessment of nociception/antinociception balance, which, when added to RE and SE, serve together as the adequacy of anesthesia (AoA) concept, a new tool (SPI; GE Healthcare, Helsinki, Finland) that has proven its utility for adequate anesthetic administration guidance, anesthetics for hypnosis, and IROA for analgesia [13], which aims to reduce the occurrence of unwelcome GA-related adverse events [14,15,16]. As the signal is derived from finger photoplethysmography, no complex preoperative preparations are required, impairing the economy of the operating theatre [17].

In our former analysis performed on a smaller group, we have observed no marked effect of PA, neither intravenous nor regional, on the perioperative outcomes in patients undergoing VRS when IROA was utilized under AoA guidance. Surprisingly, we observed a tendency toward a decreased demand for IROA using fentanyl (FNT) in two out of five groups accompanied by an equal postoperative pain relief, which raised our interest when the pre-emptive anesthesia (PA) was performed using either preoperative peribulbar block (PBB), with a mixture of 0.5% bupivacaine and 2% lidocaine, or intravenous paracetamol, with a single dose of 1 g, contrary to the topical anesthesia, using 2% proparacaine, or an intravenous metamizole, with a single dose of 1 g [18].

To the best of our knowledge, no study has been carried out in order to investigate the utility of PA PBB using different LAs or intravenous paracetamol in combination with AoA guidance for IROA administration using FNT during GA in patients undergoing VRS.

Therefore, as the expansion of the aforementioned study, the aim of the current analysis was to assess the influence of employment of PA using either PBBs using different LAs mixtures as compared to intravenous paracetamol in a single dose of 1 g under AoA guidance used for titration of rescue FNT intraoperatively on the rate of incidence of IPPP (primary outcome measure), as well as hemodynamic stability and demand of IROA (secondary outcomes) in patients undergoing VRS under GA.

## 2. Materials and Methods

This study methodology was built upon the work conducted in our previous papers [18,19,20,21].

The study included patients eligible for elective primary vitreoretinal surgery (VRS) at the Department of Ophthalmology, St. Barbara 5th Regional Hospital in Sosnowiec, Poland, who met the specific inclusion criteria. A total of 185 patients with an American Society of Anesthesiologists (ASA) score between I and III participated in this prospective, randomized, controlled trial after providing written informed consent. Randomization was conducted using sealed envelopes, assigning patients into four equal groups. The study received approval from the Bioethical Committee of the Medical University of Silesia, in accordance with the Declaration of Helsinki (approval number KNW/0022/KB1/101/15) on 29 September 2015 (Chairman: Dr. Bogusław Okopień, Polish National Consultant for Pharmacy and Clinical Pharmacology). Additionally, the trial was registered in the Clinical Trial Registry (Silesian MUKOAiIT8, NCT03413371).

In this study, exclusion criteria were history of allergy to local anesthetics or paracetamol, drug or alcohol abuse, history of neurological disease or neurosurgical procedure that could interfere with entropy EEG monitoring, cardiac arrhythmia on ECG that could interfere with SPI monitoring, history of pulmonary disease that possesses a risk of bronchospasm, symptoms suggesting difficult laryngeal mask placement, history of acute or chronic pain, pregnancy, and anti-platelet therapy that is a contraindication to PBB.

During the preoperative visit, which took place the day before vitreoretinal surgery (VRS), patients were informed about the potential for immediate postoperative pain perception (IPPP). They were instructed on how to use the 10-point numeric pain rating scale (NPRS) to report pain levels, where a score of zero indicated no pain and a score of ten represented the worst pain imaginable [18,19,20,21,22].

### 2.1. Anesthesia Technique

All patients fasted for at least 12 h due to potential diabetic gastropathy. On the day of the surgery, before the start of anesthesia, they received medication at a dose of 3.75–7.5 mg in a single dose of midazolam (Midanium, Polfa Warszawa, Poland), depending on the age and body weight administered intravenously [23].

In the BL group, upon arrival to the operating room, patients received general anesthesia (GA) along with preventive topical analgesia, which included three applications of 2% proparacaine (Alcaine), proparacaine hydrochloride ophthalmic solution, USP 0.5%, 15 mL, Sandoz, a Novartis Company, Alcon Laboratories, Fort Worth, Texas, USA). This was followed by a peribulbar block (PBB) using a mixture of 3.5 mL of 2% lidocaine (Lignocainum Hydrochloricum WZF 2% solution, 20 mg/mL, Polfa Warszawa S.A, Poland) and 3.5 mL of 0.5% bupivacaine (Bupivacainum Hydrochloricum WZF 0.5%, Polfa Warszawa S.A, Warsaw, Poland) administered via Hamilton’s technique, 15 min prior to GA induction [24].

In the BPV group, after arriving at the operating room, patients received GA alongside preventive topical analgesia by triple instillation of 2% proparacaine, followed by a PBB using 7 mL of 0.5% bupivacaine via Hamilton’s technique, 15 min before the induction of GA [24].

In the RPV group, upon arrival to the operating room, patients also received GA with preventive topical analgesia by three applications of 2% proparacaine, followed by a PBB using 7 mL of 0.75% ropivacaine (Ropivacaini Hydrochloridum 1%, 10 mg/mL, Molteni Farmaceutici, Scandicci, Italy) administered using Hamilton’s technique 15 min prior to GA induction [24].

In the P group, patients received GA 30 min before arriving at the operating room, combined with preemptive analgesia in the form of a single dose of 1 g of acetaminophen (Paracetamol Kabi, 10 mg/mL solution, Fresenius Kabi, Warsaw, Poland) mixed in 100 mL of saline [24].

Immediately before surgery, all patients were pre-oxygenated for five minutes with 100% oxygen and were intravenously administered Ringer’s solution (B. Braun Melsungen AG, Melsungen, Germany) at a dose of 10 mL/kg body weight. Anesthesia was induced intravenously using fentanyl (Fentanyl WZF, 0.1 mg/2 mL, Polpharma, Warsaw, Poland) at a dose of 1 mcg/kg, followed by a single dose of propofol at 2.5 mg/kg body weight (Propofol 1% MCT/LCT Lipuro, Fresenius Kabi GmbH, Bad Homburg, Germany) to achieve a state entropy (SE) of approximately 40–45, similar to prior studies [22,25,26,27].

Muscle paralysis was achieved in all patients using a standard intravenous dose of 0.6 mg/kg rocuronium (Esmeron, Fresenius, Warsaw, Poland) after the loss of consciousness. After 45 s, a laryngeal mask airway was placed, and CO_2_ levels were maintained at 35–37 mmHg throughout. Prior to surgery, sevoflurane was adjusted to maintain a state entropy between 35 and 45, similar to previous studies involving intravenous anesthesia during vitreoretinal surgery (VRS) [22].

Standard monitoring was implemented throughout anesthesia induction and VRS, focusing on key vital parameters such as heart rate (HR), non-invasive blood pressure (NIBP), electrocardiography (ECG) II, oxygen saturation (SaO_2_), end-tidal CO_2_ concentration (etCO_2_), fraction of inspired oxygen (FiO_2_), minimal alveolar concentration of sevoflurane (MAC), as well as the fractions of inspired and expired sevoflurane (FiAA and FeAA, respectively).

To ensure an appropriate depth of anesthesia, entropy EEG (state and response entropy) was used, and intraoperative analgesia was managed with the surgical pleth index (SPI). Neuromuscular transmission (NMT) monitoring (Carescape B650, GE, Helsinki, Finland) was also applied to maintain muscle relaxation effectively.

#### 2.1.1. Stage 1

Upon admission to the operating theater, monitoring devices were placed according to the manufacturer’s recommendations. This included positioning the EEG entropy sensors (for RE and SE) on the patient’s forehead, placing the pulse oximeter (SPI) on the finger opposite to the site of venous access, and securing the non-invasive blood pressure (NIBP) cuff on the right arm. Standard ECG electrodes were positioned on the patient’s back, and initial baseline values were then recorded.

All surgeries involving peribulbar blocks (PBB), based on group assignment, were performed by the same ophthalmic surgeon (K.K.), who has over 15 years of experience in the field of vitreoretinal surgery.

#### 2.1.2. Stage 2

In stage 2 of the procedure, to calculate the mean surgical pleth index (SPI) value, SPI measurements were collected starting from five minutes after the placement of the laryngeal mask until the beginning of orbital sterilization, allowing time for SPI sensor calibration. During this period, patients received an infusion of balanced crystalloids at a rate of 5 mL/kg/h, adjusted according to their individual needs.

#### 2.1.3. Stage 3—Intraoperatively

The surgical pleth index (SPI) was monitored continuously online, with SPI values recorded at a rate of one sample per minute. If the SPI value exceeded the mean Stage 2 SPI value by more than 15 points (∆SPI > 15), a rescue dose of fentanyl (FNT) at 1 mcg/kg body weight was administered intravenously every five minutes until the SPI value returned to the mean Stage 2 value. The duration of the vitreoretinal surgery (VRS) was measured from the installation to the removal of the speculum.

It was initially assumed that a fentanyl dose of 1 mcg per kg of body weight would provide adequate analgesia before speculum installation. Previous work by Gruenewald et al. [28] suggested that a ∆SPI > 10 or an absolute SPI value > 50 could predict inadequate analgesia. Other studies have indicated that an absolute SPI value > 50 is the threshold for administering rescue analgesia [29]. To mitigate the risk of potentially dangerous fentanyl overdose due to misinterpretation of SPI variations, a protocol was adopted where ∆SPI > 15 compared to the baseline Stage 2 value, sustained for at least one minute, was used as an indicator for rescue analgesia.

All VRS procedures were performed by the same ophthalmic surgeon (A.L-B), who has over 10 years of experience in vitreoretinal surgery, performing more than 400 procedures annually.

Intraoperative oculocardiac reflex (OCR) was monitored and was typically identified by a rapid decrease in heart rate of 20% or more from the baseline during ocular manipulations. In the event of OCR, the surgeon was instructed to temporarily stop manipulation within the surgical field. If the OCR did not resolve, 0.5 mg of atropine (Atropinum sulfuricum; Polfa, Warsaw, Poland) was administered intravenously. Additionally, if the heart rate dropped below 45 beats per minute, a rescue dose of 0.5 mg atropine was given.

For intraoperative hypotension, defined as mean arterial pressure (MAP) < 65 mmHg [30], a rescue bolus of balanced crystalloids (5 mL/kg) was infused. If MAP did not return to the normal range, 10 mg of ephedrine (Ephedrinum hydrochloricum; Polfa, Warsaw, Poland) was administered intravenously to restore normotension. Conversely, in cases of elevated MAP (>110 mmHg), urapidil (Ebrantil 25; Takeda Pharma, Vienna, Austria) was administered intravenously, provided SPI values indicated adequate anti-nociception, until MAP was normalized.

#### 2.1.4. Stage 4—Postoperatively

All patients were monitored in the post-anesthesia care unit (PACU) by anesthesia teams who were unaware of the patients’ group allocations, ensuring unbiased observation. Monitoring included surgical pleth index (SPI), heart rate (HR), systolic arterial pressure (SAP), mean arterial pressure (MAP), diastolic arterial pressure (DAP), and arterial oxygen saturation (SaO_2_). Along with these postoperative hemodynamic parameters, adverse events such as nausea, vomiting (PONV), sedation levels, allergic reactions, and pain were monitored for 24 h.

For managing PONV, a single intravenous dose of 4 mg ondansetron (Ondansetron Accord, Accord Healthcare Limited, London, UK) was given. If MAP fell below 65 mmHg, Optylite solution (Optylite, 500 mL, Fresenius Kabi SP. Z.o.o, Warsaw Poland) was administered at a dose of 5 mL/kg body weight. Patients were provided oxygen at a rate of 3 L/min via a nasal cannula. Pain intensity was assessed every 10 min using the numeric pain rating scale (NPRS), with scores ranging from 0 (no pain) to 10 (worst imaginable pain). If a patient reported pain with an NPRS score greater than 3, an appropriate dose of a non-steroidal anti-inflammatory drug (NSAID) or metamizole (MTM) was administered, in line with individual needs and contemporary guidelines for acute pain management from the Polish Society of Anaesthesiologists [6].

SPI values were recorded online, with mean values logged at a one-minute sampling rate (utilizing software provided by the manufacturer, CARESCAPE Monitor B650 software version 3.1). Pain intensity (NPRS) and SPI values were recorded for different ranges of pain perception: mild pain (NPRS 0–3), moderate pain (NPRS 4–6), and severe pain (NPRS 7–10).

Patients were observed and monitored in the PACU for at least 30 min before being transferred to the Department of Ophthalmology (DO). Upon transfer, general monitoring and data recording were stopped, except for cases involving PONV. Any occurrences of PONV during the first 24 h postoperatively were recorded, and intravenous anti-emetic treatment was administered each time it occurred. This included 4 mg of ondansetron (Ondansetron Accord, 2 mg/mL solution, Accord Healthcare Limited, London, UK) and 4 mg of dexamethasone (Dexaven, 4 mg/mL, Jelfa, Jelenia Góra, Poland). All patients were discharged to DoVS when they met 4 conditions: postoperative pain perception at rest NPRS < 4, MAP > 65 mmHg, HR > 60 or < 90 beats/minute, Aldrete score > 8.

### 2.2. Statistical Analysis

G*Power 3.1.9.7 was used to perform sample size calculations [30]. A one-way ANOVA based on the moderate effect size (f = 0.25), an alpha level of 0.05, a power of 0.8, and divided into four groups was used to estimate the sample size of 180. As data from 174 of the 185 enrolled patients were finally analyzed, a post hoc test was performed, which showed that with this sample size the power (1-β) was 0.79.

The statistical analysis was conducted using STATISTICA 13.3 (StatSoft, Kraków, Poland). The Shapiro–Wilk test was applied to verify the normality of data distribution, confirming that the data did not follow a normal distribution. Consequently, the Kruskal–Wallis test was utilized for comparisons, followed by Dunn’s post hoc test for further analysis. Quantitative data were summarized by presenting the mean, standard deviation, median, and interquartile range. For qualitative data, the chi-square test was employed, and results were reported in percentages. A *p*-value of less than 0.05 (*p* < 0.05) was considered statistically significant, indicating that the observed differences were unlikely to have occurred by chance and suggesting a meaningful effect in the analysis.

## 3. Results

**Figure 1:**
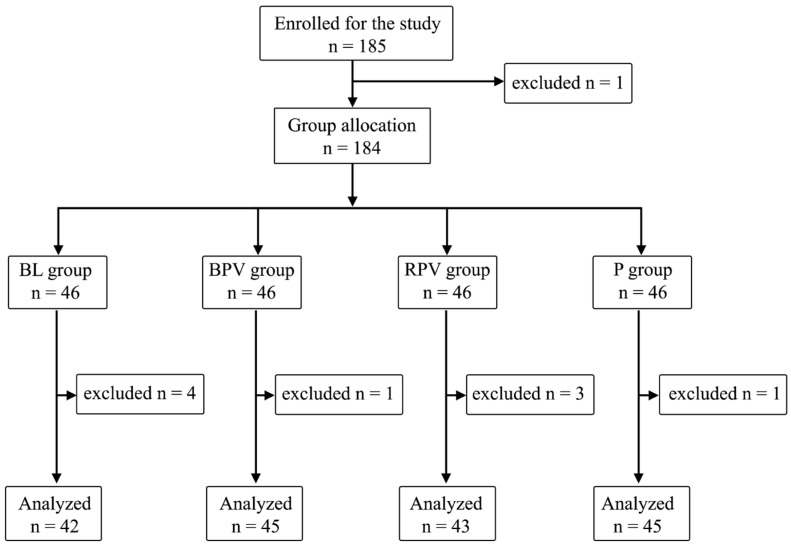
Randomization graph.

The study included 185 patients. One patient refused to participate in the morning before VRS, the latter ones were divided into four equal groups: BL, BPV, RPV, and P—46 patients (25%). Additional patients were excluded due to their refusal to participate prior to PBB at the sight of a needle—three patients; technical problems with intraoperative SPI measurement—two patients; postoperative arousal leading to inability of observation during stage 4—two patients; or an inability to declare postoperative pain perception—two patients, and expecting GA only. A total of 175 patients data were ultimately analyzed: 76 men (54.3%) and 99 women (45.7%) (Figure 1).

The detailed characteristics of patients’ anthropometric data are shown in Table 1. No significant differences in individual groups in the case of characteristics: age, height, weight, and BMI were registered.

In Table 2, the frequency of surgical maneuvers during the procedure is presented.

Staining agent injections and peelings were performed more frequently in the BPV group compared to the BL group. In the case of indentation, it was the most commonly performed in the P group, especially in relation to the BPV and RPV groups.

Table 3 shows information regarding the procedure and occurrence of postoperative pain.

**Figure 2:**
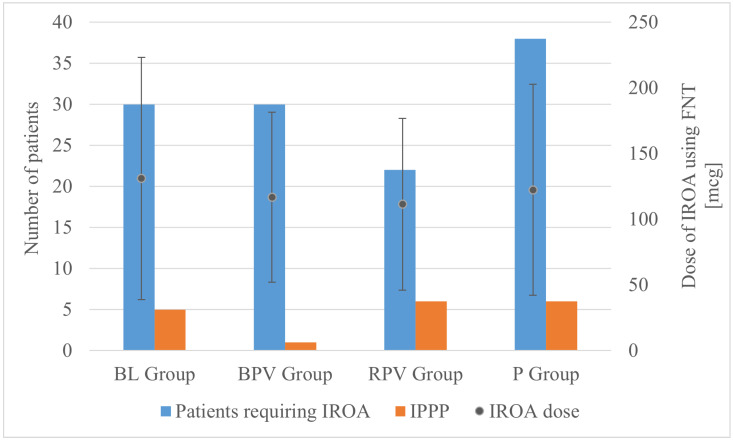
Number of patients requiring IROA (left axis), dose of IROA (right axis), and incidence of IPPP (left axis), according to group allocation. BL—bupivacaine/lidocaine group; BPV—bupivacaine group; RPV—ropivacaine group; P—paracetamol group; FNT—fentanyl; IPPP—intolerable postoperative pain perception; IROA—intraoperative rescue opioid analgesia.

The operating time was significantly longer in the BL group compared to the BPV group, whereas a difference of several minutes did not bear clinical relevance. There were no significant differences in the FNT dose. However, it was observed that significantly more patients required IROA in the P group compared to the RPV group (Figure 2). The lowest value of intraoperative fluid therapy was also noted in the P group compared to the BL and RPV groups, which can be explained by the fact that preoperatively in the DO patients allocated to the P group received paracetamol intravenously in a volume of 100 mL. If this volume is added into the volume of intraoperative fluid therapy, then the statistical significance would presumably have disappeared.

Table 4 presents hemodynamic changes in different stages of anesthesia.

No significant hemodynamic changes were noted in stage 1. In stage 2, mean SAP and MAP values were statistically significantly higher in the P group as compared to the RPV group. Similar changes were noted in stage 3 for mean SAP, MAP, and DAP values, but one must always keep in mind that mean values of arterial blood pressure yield a bias up to even, 5.5  ±  9.3 mmHg, when non-invasive intermittent technique is employed [31].

In stages 2 and 3, the mean SPI values were significantly higher in the P group compared to the RPV group, which bears little clinical relevance because when IROA using FNT boluses of 1.0 µg/kg is administered to maintain the SPI value, the SPI target is set between 20 and 50 [32], with the best target of intraoperative mean SPI value around 29 [33], which was almost achieved in the current study (see mean SPI values according to group allocation). These targets were published several years after the current study was started.

In stage 4, significant differences were only noted between the BPV and P groups for mean SPI values. What is even more surprising is that statistically significantly lower mean SPI values during stage 3 result in statistically significantly higher values during stage 4 between the same groups; that fact requires further analysis of why lower intraoperative SPI values result in higher SPI postoperative values, which completely does not correspond with the number of patients reporting IPPP in these groups, despite the lack of statistical significance between them.

In Table 5, hemodynamic fluctuations in studied groups were presented.

In stage 2, significantly higher minimum SAP, MAP, and SPI values were observed in the P group compared to the RPV group. Additionally, minimum SE was the highest in the BL group, especially in relation to the BPV and RPV groups. In stage 3, maximum SAP and MAP values were significantly higher in the P group as compared to the BPV and RPV groups. Maximum HR, maximum SPI, and minimum SE were the highest in the BL group.

In stage 4, the highest maximum SPI values were observed in the BPV group as compared to the BL and P groups, which seems to have little clinical value as maximum SPI values presumably reflect patients arousal in the PACU, whereas at that time SPI values were proven predictive of IPPP only if obtained before patients’ arousal [34]. The only reasonable explanation could be found that maximum and minimum SPI values were the lowest in the P group, compared to the other groups, where PBBs were not performed, which could have been provoked by some eye discomfort. Nevertheless, patients’ satisfaction from performed anesthesia, according to allocation to groups, was not studied, so our observation must stay as an assumption.

## 4. Discussion

The current study analysis covering the utility of PA using either intravenous paracetamol in a single dose of 1 g or peribulbar block using different LAs in patients undergoing VRS revealed the incidence of IPPP in 18 out of 175 patients (10.23%) despite the allocation to groups, similarly to our recently published analysis, where IPPP was noted in 16 out of 153 (10.46%) patients receiving different intravenous PA undergoing AoA-guided GA for VRS [22]. Our remarkable observation of only 2 patients (1.14%), one allocated to the paracetamol group and the other one to the ropivacaine group, experiencing acute postoperative pain (NPRS > 6) seems to be quite an achievement in view of the fact that the 116 (66.3%) patients received endolaser treatment and 47 (26.9%) indentation, which were already proven as the most noxious surgical maneuvers during VRS in our other study from this field [21].

No statistically significant differences were observed between groups in terms of the rate of incidence of IPPP as well as acute, moderate, and mild pain perception. Despite the allocation to groups, mean maximum postoperative pain intensity using NPRS was defined at 0.87 ± 1.76, with a statistically significant difference between patients receiving BPV (0.09 ± 0.6) and paracetamol (1.27 ± 1.97) (*p* = 0.03; *p* < 0.05) bearing little clinical significance when taking absolute numbers of patients with IPPP into consideration. Despite the group allocation, AoA guidance resulted in the necessity of IROA administration in 120 out of 175 patients (68.57%). A statistically significant difference between the absolute number of patients requiring IROA was observed between the paracetamol group—38 out of 45 (84.44%) and the ropivacaine group—22 out of 43 (51.16%); (RPV vs. P, *p* = 0.002; *p* < 0.05), which similarly did not lead to the statistically significant difference in IPPP.

The approach to anesthesia for patients undergoing vitreoretinal surgeries (VRS), which typically involves pars plana vitrectomy or facovitrectomy, sometimes combined with scleral buckling, has shifted significantly since the 1990s. Initially relying on traditional general anesthesia (GA), it has evolved into a broader use of regional anesthesia (RA) techniques in recent years [1]. However, in certain elderly patients, due to contraindications for using RA with only monitored anesthesiological care, proper immobilization on the operating table necessitates the continued use of GA, particularly for longer VRS procedures [2,3]. Coexisting conditions, the need for antiplatelet therapy, and the risk of respiratory complications during VRS under RA with monitored anesthesiological care can hinder patient cooperation, making the use of RA increasingly challenging. Thus, employing a GA regimen that includes intraoperative remifentanil opioid analgesia (IROA) encourages exploring additional preemptive analgesia (PA) options, as these have been shown to reduce overall anesthesia requirements.

Administration of IROA in every individual incident of rapid raise of heart rate and blood pressure at least > 30%, that are perceived as the indirect indicators of potentially insufficient analgesia. IPPP, hemodynamic instability, oculocardiac and oculoemetic reflex, and postoperative nausea and vomiting are the most frequent, and the incidence of the last one, often triggered by IPPP, according to some authors, may even lead to the destruction of the result of the performed VRS by triggering a rapid blood pressure increase with a subsequent subretinal hemorrhage.

Although there are conflicting data in terms of the impact of paracetamol and PBB using either lidocaine, or bupivacaine or ropivacaine or solutions containing their mixtures with different proportions and concentrations on the dose of IROA potential sparring effect and reduction of IROA dosing-related rate of incidence of adverse events, they were proven to possess analgesic efficacy as a decent part of a multimodal concept prior to minor-to-intermediate surgery being widely employed to achieve satisfactory improvement of postoperative pain relief [35,36,37,38].

Ropivacaine and bupivacaine, contrary to short-acting amide-type and neurotoxic lidocaine [39], are both long-acting, amide-type local anesthetics with such a difference that ropivacaine is a pure S(−)-enantiomer, whereas bupivacaine is a racemate. PBB with ropivacaine provides strong analgesia during a longer operation time with reduced severity of neurotoxicity as compared to lidocaine and reduced severity of cardiotoxicity as compared to bupivacaine [40,41].

Numerous studies have proven that the combination of PA with GA may reduce the demand for IROA administration.

Schönfeld et al. have proven the utility of PBB as a renowned part of the general concept of PA by performing the peribulbar injection of 5 mL of 0.75% ropivacaine before ophthalmic surgery that provided a substantial benefit in terms of analgesic demand and postoperative comfort in recovery [10], likewise in the study by Ghali et al., where the performance of PBB using the same LA mixture as in the BL group in the current study protocol in conjunction with GA but with no AoA guidance for IROA administration proved superior to GA alone for VRS in terms of reduction of rate of incidence of GA-related adverse events [5]. Similarly, Jaichandran et al. have proven that 0.5% bupivacaine solution used for PBB—the same LA mixture as in the BPV group in the current study protocol—provides better quality of analgesia as compared to LA mixture of 2% lidocaine and 0.5% bupivacaine—the same LA mixture as in the BPV group in the current study protocol—in patients undergoing VRS [42]. More importantly, adequate anesthesia and akinesia (grade 5) was achieved in 56.7% of the patients in the BPV group as compared to only 23.3% in the lidocaine group and 30% in the combination BL group, which supports our regimen of combined general/regional anesthesia under AoA guidance because, in some cases, proper akinesia is not achievable by PBB alone. Similarly, Subramanian et al. noted the necessity of supplemental IROA in 16.7% of patients receiving PBB using 0.75% ropivacaine [43], better in the current study, but they based it on patients’ subjective perceptions.

Jaichandran et al. in another study observed that 0.75% ropivacaine—the same LA mixture like in the BPV group in the current study protocol—was a choice of a better quality of LAs mixture for patients undergoing VRS as compared to 0.5% bupivacaine [44], similarly to Luchetti et al., who concluded that PBB using ropivacaine—the same LA mixture like in the BPV group in the current study protocol—provided superior over a bupivacaine–mepivacaine mixture, with reduced pain and need for top-up injections [45]. When taking intravenous PA into consideration, paracetamol has already been well recognized due to its analgesic efficacy, and as a result, is widely used in the prevention of the incidence of IPPP [36,46] and also in patients undergoing VRS [47]. Paracetamol (acetaminophen) is one of the two most widely used non-opioid analgesics that possesses both a central (inhibition of cyclooxygenase type 3—COX-3) and peripheral mechanism of action [48,49,50,51,52,53]. Its analgesic efficacy in treatment of postoperative pain is observed even following administration of a single dose [35,36,54,55,56,57], results in reduction of the rate of incidence of PONV [54,57,58], enjoys a pretty good safety profile [36,55,57,59] as compared to standard non-steroid anti-inflammatory drugs (NSAIDs), in terms of protection in the upper intestinal tract and kidneys in patients with an increased risk for stomach or renal problems or other numerous contraindications for standard NSAIDs [37,55,60], Therefore, its employment as PA in the control group seemed a natural choice, as its efficacy in patients undergoing VRS has been reported but never compared with PBBs using different LAs, to the best of our knowledge. Unlike metamizole, it neither induces agranulocytosis [61], nor anaphylaxis [62], nor Kounis syndrome (coincidental occurrences of allergic reaction and acute coronary syndrome secondary to vasospasm) [63], but was reported to provoke tachycardia, hypertension, hepatotoxicity, and nephrotoxicity [55]. These were not observed in patients in the current study, although its administration proves inferior to PBBs in terms of analgesia quality, as expressed by the number of patients requiring IROA, especially during the indentation stage in the current study.

Employment of different PA modalities did not lead to complete elimination of demand for IROA, which administration has been gaining increasing popularity under monitoring of nociception/anti-nociception balance. Digital monitoring of intraoperative analgesia efficacy by observance of nociception defined as surgical painful stimulation and anti-nociception—intraoperative rescue analgesia regardless of its type is therefore gaining increasing popularity [64,65]. Under a condition of stable hypnotic component expressed by SE within a range of 40–45, the control of the intraoperative efficacy of IROA administration during GA using surgical pleth index (SPI), reflecting the abovementioned balance [28], was proven to be more effective as compared to IROA administration based on observation of hemodynamic changes in reaction to painful stimuli intraoperatively [66,67,68]. Observance of the SPI value increase on the screen in a digital form (0—no painful stimulation, 100—maximum painful stimulation) and in graphic form as a ball jumping up and down after a painful stimulus and returning to a baseline level after IROA bolus makes the monitoring of IROA titration intuitive and reliable [69] because SPI values fluctuate in response to nociception correlated with serum opioid concentration [70]. During general anesthesia (GA), insufficient intraoperative analgesia can result in painful (nociceptive) stimuli from the surgical field, which in turn triggers the release of stress hormones, leading to an increase in heart rate and arterial blood pressure. The administration of intraoperative remifentanil opioid analgesia (IROA) can effectively mitigate these effects through anti-nociception. However, excessive dosing of IROA may result in significant hemodynamic instability, causing bradycardia and hypotension and necessitating an increased need for intravenous fluid administration to manage these adverse effects. 

Numerous countermeasures are also directed to predict and prevent the incidence of major adverse cardiovascular events after noncardiac surgeries [71]. This is especially important for high-risk patients in whom either IROA or LAs are used because they may both bear responsibility for their appearance. Although cardiac failures are mainly observed following higher doses of LAs than those used in PBB [72], hypotension and bradycardia were observed in patients receiving PBB using ropivacaine in the volumes and concentrations used in the course of the current study [73], although it is marketed as dedicated for patients with cardiovascular co-morbidities. Intraoperative hypotension, which is defined as MAP ≤ 65 mmHg [74], supposedly dangerous when lasting at least 15 min, in the current study was not observed, because mean MAP values, regardless of allocation to groups intraoperatively, did not meet the abovementioned criteria of intraoperative hypotension (stage 3), and minimum MAP values did not also fall below the abovementioned norm, being higher than 65 mmHg, which was not statistically significant. During stage 3, mean SAP, MAP, and DAP values were observed within a clinical norm [75]. Observed in the current study, statistically significant differences bear little clinical relevance with a little preference for the P group, presenting better hemodynamic stability, as compared to patients receiving PBBs with different mixtures of LAs, as fluctuations of mean arterial blood pressure values between stages 1 to 4 of the study seemed to be less wavy.

In our opinion, the perioperative effects of hemodynamic stability and rate of incidence of IPPP achieved in our study analysis could possibly be explained by the employment of SPI-guided IROA titration and comparable suppression of brain function by the employment of entropy EEG between individual patients to administer the hypnotic component of GA (reaching the proper depth for anesthesia following a bolus of propofol in a single dose of 2.5 mg/kg of body weight followed by the precise administration of volatile sevoflurane—(see mean SE values at stages 2 and 3 in Table 4), that both form the AoA monitoring concept of GA.

The precision of administration of IROA (see mean SPI values at stages 2 and 3 in Table 4) only in the case of ineffective intraoperative analgesia as defined by ∆SPI > 15 and stable hypnosis as defined by SE around 40–45 (during stage 3 mean SE values were not statistically significantly different between groups) has probably led to a stable FNT serum concentration, as fluctuations of SPI values were proven to correspond to opioid serum concentration [70] and supposedly resulted in effective suppression of afferent nociceptive stimulation responsible for evoking the phenomenon of IPPP. The employment of SPI to guide IROA administration using FNT supposedly prevented possible induction of IPPP in the mechanism of suppression of central sensitization [7], whose role has already been well-described in the process of triggering IPPP, as was similarly observed in the study of Jain et al. [76]. Additionally, we assume that stable intraoperative analgesia achieved by employment of SPI-guided IROA administration effectively completed the effect of employment of a PA regimen using either paracetamol being an intravenous COX-3 inhibitor or widely used LAs mixtures for PBB at most noxious stages (trocar insertion, vitrectomy maneuvers, indentation, endolaser treatment, subconjunctival injection) in each individual case and therefore formed the efficient anti-nociception together with stable hemodynamics, reducing the rate of incidence of evoked IPPP by surgical manipulations in the operation site during VRS, likewise in our former study [18]. As PBBs, regardless of mixture of Las, have proven no advantage over intravenous paracetamol, we recommend applying AoA guidance for IROA administration during GA in patients undergoing VRS with combined PA using both paracetamol and metamizole, based on our findings from two former projects, where we have proven superiority of AoA guidance for IROA administration with COX-3 cyclooxygenase inhibitors as PA to reduce the rate of incidence of IPPP [22] and reduce the rate of incidence of PONV when separately used [18]. It is worth underlining that in our recently published study in a group of patients receiving combined PA using metamizole and paracetamol, only 1 out of 53 patients (1.88%) experienced IPPP (1.88%), whereas in the present study only in patients receiving bupivacaine alone as PA, such a result was nearly achievable (2.22%), accompanied by all the risk involved in the performance of PBB that intravenous PA is free from. Therefore, we find the utility of preventive PBBs questionable in terms of possible, although seldom observed, serious complications connected with them as well as the impairment of economic frugality, when the possibility of AoA guidance for IROA employment is at hand.

There are several limitations to the current analysis. Firstly, each incidence of IPPP is a subjective phenomenon, whose quantification is burdensome [77]. The study did not include a control group with no AoA-guided administration regimen on purpose, as numerous similar studies have already been performed and the findings are widely available [5,10], and monitoring of nociception/anti-nociception balance has become a decent standard in clinical practice [65,78,79], although some studies still doubt its efficacy [80]. Similarly, we abandoned the idea of the ropivacaine/lidocaine group, which some might find questionable, whereas a mixture of two LAs producing sensoric blocks and diluting them in one solution might only lead to the deterioration of sensoric block quality [81]. This may create bioethical concerns—little clinical effect of PBB with a standard danger of PBB-related adverse events [42].

We decided to analyze the rates of OCR, oculoemetic reflex, and PONV, as well as relationships between SPI, values of intraoperative maneuvers, and patients’ NPRS, as separate additional studies because the manuscript is approaching the word count limit.

We found it futile to analyze the rate of IPPP after discharge from PACU to the DO on purpose as the project involved monitoring NPRS as well as SPI values in stage 4, whereas patients arousal (changing position in bed, cough, etc.) markedly interferes in SPI monitoring [34]; therefore, such a comparison is laborious and of limited clinical value. Finally, a single dose of paracetamol and PBB, depending on the LA mixture, are both known to act up to several hours, so comparison of NPRS values over a longer period of time bears little clinical relevance.

## 5. Conclusions

In our analysis, a limited advantage of intravenous infusion of either 1 g paracetamol or performance of PBBs using different LAs prior to VRS was observed in terms of rate of incidence of IPPP and hemodynamic stability, contrary to the conclusions from numerous literatures. Surprisingly, PBB using ropivacaine diminished the number of patients requiring IROA, whereas PBB using bupivacaine diminished the maximum NPRS in the PACU, in both cases with no influence of demand for its dose, as compared to paracetamol, but their use did not influence perioperative outcomes. We hypothesize that the precision of administration of IROA using FNT under AoA guidance resulted in suppression of central sensitization of a decent quality and therefore supposedly blunted the impact of the use of PA. We recommend applying AoA guidance for IROA administration during GA in patients undergoing VRS as standard monitoring based on findings from our three completed projects.

## Figures and Tables

**Table 1:** Anthropometric data in groups of patients studied.

Metrics		Total *n* = 175 (100%)	BL *n* = 42 (24%)	BPV *n* = 45 (25.71%)	RPV *n* = 43 (24.57%)	P *n* = 45 (25.71%)	*p*-Value
Age X ± Sd Me (IQR)	[years]	66.1 ± 9.5 67 (11)	66.3 ± 11.4 66.5 (13)	66.29 ± 7.6 68 (8)	66.23 ± 9.7 68 (12)	65.24 ± 9.6 66 (9)	0.83 NS
Gender *n* (%)	Female	99 (56.57)	26 (61.9)	23 (51.1)	19 (44.2)	31 (68.9)	0.09 NS
	Male	76 (43.43)	16 (38.1)	22 (48.9)	24 (55.8)	14 (31.1)	
Anthropometric Data		Total *n* = 175 (100%)	BL *n* = 42 (24%)	BPV *n* = 45 (25.71%)	RPV *n* = 43 (24.57%)	P *n* = 45 (25.71%)	*p*-Value
Height X ± Sd Me (IQR)	[cm]	166.2 ± 9 165 (13)	165.7 ± 8.3 164.5 (10)	168.9 ± 9 168 (11)	166.7 ± 9.94 168 (18)	163.7 ± 8.1 163 (11)	0.07 NS
Weight X ± Sd Me (IQR)	[kg]	77.4 ± 14.4 76 (20)	77.3 ± 16.1 73 (14)	80.3 ± 13.3 79 (21)	78.1 ± 15 76 (19)	74.2 ± 13.1 74 (19)	0.22 NS
BMI X ± Sd Me (IQR)	[kg/m^2^]	28 ± 4.7 27.7 (5.9)	28.2 ± 5.5 26.8 (4.8)	28 ± 3.3 27.5 (4.9)	28.1 ± 4.7 28.1 (7)	27.8 ± 5 27.9 (7)	0.86 NS
BMI *n* (%)	norm	51 (30)	12 (29.3)	9 (22)	14 (32.6)	16 (35.6)	0.6 NS
	overweight	69 (40.6)	20 (48.8)	19 (46.3)	15 (34.9)	15 (33.3)	0.4 NS
	obesity	50 (29.4)	9 (22)	13 (31.7)	14 (32.6)	14 (31.1)	0.7 NS

BL—bupivacaine/lidocaine group; BPV—bupivacaine group; RPV—ropivacaine group; P—paracetamol group; Sd—standard deviation; Me—median; IQR—interquartile range; BMI—body mass index; kg—kilogram; m—meter; cm—centimeter; NS—not significant.

**Table 2:** The frequency of surgical maneuvers performed during the procedure was documented in terms of both numbers and percentages.

Type of VRS	Total *n* = 175 (100%)	BL *n* = 42 (24%)	BPV *n* = 45 (25.71%)	RPV *n* = 43 (24.57%)	P *n* = 45 (25.71%)	*p*-Value
Pars Plana Vitrectomy Yes (%)	62 (35.4)	18 (42.9)	14 (31.1)	12 (27.9)	18 (40)	0.41 NS
Phacovitrectomy Yes (%)	113 (64.6)	24 (57.1)	31 (68.9)	31 (72.1)	27 (60)	0.41 NS
The frequency of surgical maneuvers during VRS						
Speculum Adjustment Yes (%)	175 (100)	42 (100)	45 (100)	43 (100)	45 (100)	**-**
Trocars’ Insertion Yes (%)	175 (100)	42 (100)	45 (100)	43 (100)	45 (100)	**-**
Vitrectom Insertion Yes (%)	28 ± 4.7 27.7 (5.9)	28.2 ± 5.5 26.8 (4.8)	28 ± 3.3 27.5 (4.9)	28.1 ± 4.7 28.1 (7)	27.8 ± 5 27.9 (7)	0.86 NS
Staining Agent Injection Yes (%)	155 (88.6)	34 (81)	44 (97.8)	37 (86)	40 (88.9)	BL vs. BPV, *p* = 0.03
Peeling Yes (%)	155 (88.6)	34 (81)	44 (97.8)	37 (86)	40 (88.9)	BL vs. BPV, *p* = 0.03
Gas-Liquid Exchange Yes (%)	52 (29.7)	13 (31)	11 (24.4)	10 (23.3)	18 (40)	0.29 NS
Endolaser Treatment Yes (%)	116 (66.3)	28 (66.7)	29 (64.4)	28 (65.1)	31 (68.9)	0.97 NS
Silicon Oil Injection Yes (%)	42 (24)	8 (19)	10 (22.2)	11 (25.6)	13 (28.9)	0.73 NS
Indentation Yes (%)	47 (26.9)	14 (33.3)	3 (6.7)	6 (14)	24 (53.3)	BL vs. BPV, *p* = 0.004; BPV vs. P, *p* < 0.001; RPV vs. P, *p* < 0.001
Subconjunctival Injection Yes (%)	175 (100)	42 (100)	45 (100)	43 (100)	45 (100)	-
Trocars’ Removal Yes (%)	175 (100)	42 (100)	45 (100)	43 (100)	45 (100)	-
Speculum Removal Yes (%)	175 (100)	42 (100)	45 (100)	43 (100)	45 (100)	-

VRS—vitreoretinal surgery; BL—bupivacaine/lidocaine group; BPV—bupivacaine group; RPV—ropivacaine group; P—paracetamol group; NS—not significant.

**Table 3:** Characteristics of the performed treatments in groups and the assessment of occurrence of postoperative pain in the examined groups.

VRS		Total *n* = 175 (100%)	BL *n* = 42 (24%)	BPV *n* = 45 (25.71%)	RPV *n* = 43 (24.57%)	P *n* = 45 (25.71%)	*p*-Value
Time of VRS X ± Sd Me (IQR)	[min]	51.32 ± 18.32 48 (25)	59.4 ± 22.74 59.5 (38)	45.73 ± 15.38 43 (18)	47.74 ± 16.05 43 (24)	52.78 ± 15.93 51 (22)	BL vs. BPV, *p* = 0.01
Dose of IROA using FNT X ± Sd Me (IQR)	[mcg]	121.08 ± 76.78 100 (100)	131 ± 92.12 100 (150)	116.67 ± 64.77 100 (100)	111.36 ± 65.34 100 (100)	122.37 ± 80.28 100 (100)	0.97 NS
Number of patients requiring IROA	Yes (%)	120 (68.57)	30 (71.43)	30 (66.67)	22 (51.16)	38 (84.44)	RPV vs. P, *p* = 0.002
Intraoperative fluid therapy X ± Sd Me (IQR)	[mL]	1082.85 ± 309.42 1000 (350)	1097.56 ± 270.64 1000 (250)	1057.21 ± 284.14 1000 (450)	1234.39 ± 355.48 1250 (500)	940 ± 254.75 1000 (250)	BL vs. P, *p* = 0.04; RPV vs. P, *p* < 0.001
Postoperative pain perception in the PACU							
NPRS max X ± Sd Me (IQR)	[1 ÷ 10]	0.87 ± 1.76 0 (0)	0.95 ± 1.78 0 (1)	0.09 ± 0.6 0 (0)	1.19 ± 2.07 0 (2)	1.27 ± 1.97 0 (2)	BPV vs. P, *p* = 0.03
Type of first postoperative pain perception *n* (%)	Mild	157 (89.2)	37 (88.1)	44 (97.78)	37 (86.05)	39 (84.78)	0.22 NS
	Moderate	16 (9.09)	5 (11.9)	1 (2.22)	5 (11.63)	5 (10.87)	0.17 NS
	Acute	2 (1.14)	0 (0)	0 (0)	1 (2.33)	1 (2.17)	1.0 NS
	IPPP	18 (10.23)	5 (11.9)	1 (2.22)	6 (13.96)	6 (13.04)	0.22 NS

VRS—vitreoretinal surgery; BL—bupivacaine/lidocaine group; BPV—bupivacaine group; RPV—ropivacaine group; P—paracetamol group; Sd—standard deviation; Me—median; IQR—interquartile range; NS—not significant; FNT—fentanyl; NPRS—numerical pain rating scale; IPPP—intolerable postoperative pain perception; IROA—intraoperative rescue opioid analgesia; PACU—postoperative care unit.

**Table 4:** Hemodynamic changes in patients with intraoperative pain perception during certain stages of anesthesia.

Parameter	BL *n* = 42 (24%)	BPV *n* = 45 (25.71%)	RPV *n* = 43 (24.57%)	P *n* = 45 (25.71%)	*p*-Value
Stage 1—onset					
SAP (mmHg)	146 ± 26.63 143 (35)	151.64 ± 17.79 152 (27)	151.91 ± 18.56 151 (25)	153 ± 19.2 157 (28)	0.5 NS
MAP (mmHg)	108.57 ± 15.3 108.5 (24)	112.47 ± 13.13 111 (18)	109.63 ± 10.66 109 (15)	110.53 ± 12.21 111 (17)	0.68 NS
DAP (mmHg)	79.26 ± 9.59 79 (14)	83.6 ± 10.85 82 (14)	80.16 ± 7.86 80 (11)	78.42 ± 10.66 76 (15)	0.14 NS
HR (beats/min)	70.64 ± 11.92 72.5 (19)	70.24 ± 9.48 69 (17)	70.56 ± 10.18 68 (17)	73.89 ± 11.23 74 (14)	0.38 NS
SPI	55.74 ± 20.09 62.5 (32)	59.42 ± 16.78 65 (25)	58.7 ± 14.96 61 (24)	55.58 ± 16.27 51 (24)	0.6 NS
Stage 2—between GA induction and start of VRS					
mean SAP (mmHg)	124.74 ± 24.76 119.5 (32)	129.39 ± 26.83 129 (38.3)	120.1 ± 23.49 115.7 (34)	134.87 ± 25.92 134 (40)	RPV vs. P, *p* = 0.03
mean MAP (mmHg)	92.34 ± 16.91 88.8 (23)	96.07 ± 17.75 94.5 (23)	89.49 ± 15.83 88 (20)	98.43 ± 16.87 100 (26.5)	RPV vs. P, *p* = 0.048
mean DAP (mmHg)	70.4 ± 11.91 69 (18.3)	74.03 ± 12.61 73 (20)	68.34 ± 12.47 67 (15)	72.86 ± 11.78 73 (17.3)	0.12 NS
mean HR (beats/min)	69.73 ± 11 68.2 (18.5)	69.55 ± 9 68.8 (12)	66.67 ± 11.51 66.4 (16.3)	68.78 ± 9.82 68 (12.3)	0.48 NS
mean SPI	31.64 ± 9.55 29.5 (11)	33.69 ± 12.79 28.7 (14)	28.13 ± 9.83 25.7 (12.6)	39.81 ± 36.83 33 (17.1)	RPV vs. P, *p* = 0.01
mean SE	42.45 ± 8.8 42.2 (13)	39.89 ± 7.87 38.8 (13)	42.47 ± 7.63 41.4 (10.6)	40.6 ± 10.15 39.8 (16.6)	0.34 NS
Stage 3—VRS					
mean SAP (mmHg)	105.25 ± 16.51 100.2 (16.2)	100.97 ± 13.85 99.7 (21.39)	102.3 ± 20.47 95.2 (21.7)	109.76 ± 22.48 106 (24.9)	RPV vs. P, *p* = 0.04
mean MAP (mmHg)	79.81 ± 11.09 76.9 (11.9)	77.41 ± 9.48 75.1 (14.5)	77.77 ± 14.45 73.6 (15.1)	83.28 ± 12.54 81.3 (14.5)	RPV vs. P, *p* = 0.03
mean DAP (mmHg)	61.38 ± 8.53 60 (12.1)	60.43 ± 8.1 59 (11.64)	59.25 ± 11.57 57.4 (12.6)	63.11 ± 9.95 63.4 (10.5)	RPV vs. P, *p* = 0.04
mean HR (beats/min)	62 ± 8.64 62.9 (12.5)	59.26 ± 7.07 58.2 (9.65)	59.44 ± 8.25 58.3 (13.4)	60.78 ± 8.67 60.3 (12.4)	0.31 NS
mean SPI	31.74 ± 7.36 31.4 (9.1)	29.53 ± 8.18 28.3 (9.31)	27.4 ± 7.54 25.8 (8.7)	34.85 ± 10.86 31.7 (9.8)	BL vs. RPV, *p* = 0.02; BPV vs. P, *p* = 0.04; RPV vs. P, *p* = 0.001
mean SE	44.77 ± 6.33 45.5 (9.3)	42.49 ± 7.48 41.6 (6.94)	44.66 ± 6.67 45.7 (7)	41.2 ± 7.71 40.5 (10.6)	0.06 NS
Stage 4—postoperatively					
mean SAP (mmHg)	147.01 ± 18.82 143.8 (24.2)	150.68 ± 150.68 152 (18)	147.6 ± 18.41 146.8 (23.3)	150.41 ± 18.61 153.6 (29.8)	0.58 NS
meanMAP (mmHg)	102.29 ± 14.19 102.8 (13.3)	108.4 ± 108.4 110.5 (17.7)	102.8 ± 13.86 103.3 (19.3)	103.06 ± 16.12 103.3 (19.1)	0.13 NS
mean DAP (mmHg)	80.59 ± 11.81 77.3 (14.3)	82.48 ± 82.48 83.3 (14.1)	80.6 ± 14.54 77.2 (19.3)	80.17 ± 14.72 78.1 (14.7)	0.39 NS
mean HR (beats/min)	72.48 ± 11.09 71.1 (13.2)	70.08 ± 70.08 70.9 (14)	70.09 ± 9.89 70 (12.6)	68.55 ± 9.69 66.6 (13.9)	0.44 NS
mean SPI	55.91 ± 13.5 54.8 (23.2)	62.05 ± 62.05 63.4 (18.3)	58.05 ± 11.03 60.4 (18.6)	52.8 ± 15.38 52.2 (24)	BPV vs. P, *p* = 0.009

BL—bupivacaine/lidocaine group; BPV—bupivacaine group; RPV—ropivacaine group; P—paracetamol group; SAP—systolic arterial pressure; MAP—mean arterial pressure; DAP—diastolic arterial pressure; HR—heart rate; SPI—surgical pleth index; VRS—vitreoretinal surgery; GA—general anesthesia; NS—not significant.

**Table 5:** Comparison of hemodynamic fluctuations values of monitored patients’ parameters at the same stage between studied groups.

Parameter	BL *n* = 42 (24%)	BPV *n* = 45 (25.71%)	RPV *n* = 43 (24.57%)	P *n* = 45 (25.71%)	*p*-Value
Stage 2—between GA induction and start of VRS					
max SAP (mmHg)	137.36 ± 27.16 132 (33)	141.37 ± 28.43 140 (49)	131.58 ± 27.05 128 (46)	142.42 ± 25.52 147 (38)	0.13 NS
max MAP (mmHg)	101.05 ± 17.86 97 (25)	104.81 ± 19.69 104 (31)	96.81 ± 18.08 95 (28)	103.51 ± 16.01 104 (21)	0.12 NS
max DAP (mmHg)	76.14 ± 12.03 75.5 (17)	80.88 ± 14.57 78 (20)	73.58 ± 14.62 72 (22)	76.27 ± 11.55 78 (18)	0.09 NS
max HR (beats/min)	76.93 ± 13.4 75 (18)	75.22 ± 10.94 73 (13)	74.47 ± 13.03 74 (17)	74.38 ± 11.42 73 (15)	0.77 NS
max SPI	40.02 ± 10.78 39 (12)	45.91 ± 17.54 43 (33)	38.86 ± 14.68 35 (18)	43.02 ± 14.3 43 (21)	0.19 NS
max SE	48.39 ± 12.9 45 (15)	51.76 ± 16.01 48 (18)	53.68 ± 12.14 52 (12.5)	46.56 ± 10.18 45 (17)	0.08 NS
min SAP (mmHg)	114.24 ± 26.09 109 (36)	118 ± 29.72 115 (41)	109.81 ± 23.69 106 (29)	127.09 ± 28.23 133 (45)	RPV vs. P, *p* = 0.02
min MAP (mmHg)	85.74 ± 17.19 83.6 (22)	88.77 ± 18.61 86 (24)	82.98 ± 16.18 80 (22)	93.44 ± 18.62 94 (29)	RPV vs. P, *p* = 0.04
min DAP (mmHg)	65.36 ± 12.98 64.5 (15)	68.51 ± 13.4 67 (20)	63.6 ± 12.27 62 (17)	69.51 ± 12.99 67 (20)	0.15 NS
min HR (beats/min)	65.14 ± 11.18 65 (17)	64.96 ± 9.15 63 (12)	62.35 ± 11.23 61 (15)	65.27 ± 9.28 65 (11)	0.42 NS
min SPI	25.14 ± 8.54 25.5 (13)	26.33 ± 12.95 22 (12)	21.05 ± 8.21 19 (10)	28.09 ± 10.69 25 (12)	RPV vs. P, *p* = 0.002
min SE	37.01 ± 9.12 39 (11)	29.42 ± 7.8 28 (9)	30.45 ± 8.43 32 (9.5)	33.97 ± 9.69 33 (13)	BL vs. BPV, *p* < 0.001; BL vs. RPV, *p* = 0.02
Stage 3—VRS					
max SAP (mmHg)	131.36 ± 26.51 125 (38)	121.13 ± 24.22 118 (27)	120.6 ± 31.48 111 (33)	138.44 ± 26.79 135 (33)	BPV vs. P, *p* = 0.01; RPV vs. P, *p* = 0.001
max MAP (mmHg)	98.89 ± 17.29 94.2 (25)	91.36 ± 16.81 90 (20)	90.78 ± 19.98 86 (16)	103.16 ± 17.92 102 (25)	BPV vs. P, *p* = 0.01; RPV vs. P, *p* = 0.001
max DAP (mmHg)	75.52 ± 12.81 74 (20)	71.82 ± 12.38 69 (14)	70.56 ± 15.11 68 (19)	76.98 ± 13.1 75 (23)	0.06 NS
max HR (beats/min)	75.21 ± 14.37 73 (15)	66.22 ± 8.26 65 (10)	67.09 ± 10.84 65 (13)	70.29 ± 10.84 68 (18)	BL vs. BPV, *p* = 0.005; BL vs. RPV, *p* = 0.02
max SPI	53.38 ± 11.5 53 (14)	47.31 ± 13.05 47 (18)	42.77 ± 11.87 38 (15)	52 ± 12.84 53 (16)	BL vs. RPV, *p* < 0.001; RPV vs. P, *p* = 0.004
max SE	54.9 ± 6.91 54 (11)	52.69 ± 10.76 50 (14)	55.69 ± 8.49 56 (11)	54.07 ± 7.91 56 (14)	0.17 NS
min SAP (mmHg)	87.74 ± 15.85 86.5 (17)	88.6 ± 12.94 85 (15)	89.16 ± 17.03 87 (21)	90.82 ± 17.05 87 (26)	0.91 NS
min MAP (mmHg)	66.38 ± 10.76 64.5 (13)	67.91 ± 9.55 66 (10)	68.19 ± 12.37 66 (14)	68.64 ± 12.15 65 (17)	0.8 NS
min DAP (mmHg)	50.67 ± 8.18 49.5 (13)	52.78 ± 7.52 52 (9)	52.33 ± 9.99 51 (11)	52 ± 8.66 48 (14)	0.58 NS
min HR (beats/min)	55.07 ± 8.19 57 (13)	54 ± 7.04 53 (9)	54.47 ± 7.83 53 (11)	54.91 ± 8.16 54 (11)	0.92 NS
min SPI	19.36 ± 6.48 19 (8)	19.8 ± 6.69 18 (8)	18.51 ± 6.37 17 (8)	23.07 ± 8.92 22 (9)	0.06 NS
min SE	35.71 ± 8.11 36 (9)	34.04 ± 7.16 34 (7)	34.64 ± 6.84 35 (8)	31.82 ± 6.97 32 (7)	BL vs. P, *p* = 0.008
Stage 4—PACU					
max SAP (mmHg)	157.03 ± 22.44 151.5 (35.5)	160.62 ± 18.42 161 (29)	158.21 ± 21.86 157 (34)	157.84 ± 19.88 162 (31.5)	0.77 NS
max MAP (mmHg)	110.4 ± 17.06 109 (22)	116.16 ± 15.47 119 (23)	109.5 ± 15.48 108.5 (20)	108.59 ± 16.77 108 (22.5)	0.12 NS
max DAP (mmHg)	86.5 ± 11.1 84 (12.5)	88.09 ± 12.4 8 9 (14)	86.71 ± 14.28 85 (16)	85.11 ± 15.06 83 (15.5)	0.41 NS
max HR (beats/min)	77.9 ± 11.22 79 (13)	76.98 ± 11.1 77 (14)	76.81 ± 11.98 77 (17)	72.89 ± 10.83 72.5 (18.5)	0.18 NS
max SPI	64.8 ± 14.14 63 (22)	72.82 ± 13.74 76 (18.5)	69.93 ± 11.58 71.5 (14)	60.91 ± 15.58 58 (27)	BL vs. BPV, *p* = 0.03; BPV vs. P, *p* = 0.001
min SAP (mmHg)	138.03 ± 18.55 135 (17.5)	142.33 ± 14.43 143 (18)	137.17 ± 17.96 138 (31)	144.14 ± 18.9 144 (32)	0.17 NS
min MAP (mmHg)	96.19 ± 14.97 97 (19)	101.09 ± 14 105 (18)	96.39 ± 13.33 96.5 (17)	98.73 ± 17.1 100.2 (21)	0.2 NS
min DAP (mmHg)	75.9 ± 13.16 73.5 (15)	77.44 ± 11.58 78 (11)	76.31 ± 14.42 74.3 (20)	76.34 ± 15.31 74 (17.5)	0.6 NS
min HR (beats/min)	67.43 ± 10.95 65 (15)	64.13 ± 9.07 64 (15)	62.93 ± 10.27 60.5 (17)	65.23 ± 8.82 64 (13)	0.22 NS
min SPI	47.3 ± 13.59 49 (21)	50.45 ± 16.89 52 (22.5)	46.55 ± 13.09 47 (18)	45.07 ± 14.89 44.5 (23)	0.26 NS

BL—bupivacaine/lidocaine group; BPV—bupivacaine group; RPV—ropivacaine group; P—paracetamol group; SAP—systolic arterial pressure; MAP—mean arterial pressure; DAP—diastolic arterial pressure; HR—heart rate; SPI—surgical pleth index; VRS—vitreoretinal surgery; GA—general anesthesia; PACU—post-anesthesia care unit; NS—not significant.

## Data Availability

The data used to support the findings of this study are included within the article.
